# Assessment of psychological resilience in a large cohort of the general population: Validation and norm values of the RS-5

**DOI:** 10.1371/journal.pone.0309197

**Published:** 2024-10-11

**Authors:** Anna C. Reinwarth, Omar Hahad, Jasmin Ghaemi Kerahrodi, Elmar Brähler, Klaus Lieb, Donya Gilan, Daniela Zahn, Julian Chalabi, Alexander K. Schuster, Markus Schepers, Karl J. Lackner, Jörn M. Schattenberg, Wolfram Ruf, Philipp S. Wild, Andreas Daiber, Matthias Michal, Manfred E. Beutel, Thomas Münzel

**Affiliations:** 1 Department of Psychosomatic Medicine and Psychotherapy, University Medical Center of the Johannes Gutenberg-University Mainz, Mainz, Germany; 2 Department of Psychiatry and Psychotherapy, University Medical Center of the Johannes Gutenberg-University Mainz, Mainz, Germany; 3 Department of Cardiology–Cardiology I, University Medical Center of the Johannes Gutenberg-University Mainz, Mainz, Germany; 4 German Center for Cardiovascular Research (DZHK), Partner Site Rhine-Main, Mainz, Germany; 5 Department of Psychiatry and Psychotherapy, Medical Faculty, University of Leipzig, Leipzig, Germany; 6 Leibniz Institute for Resilience Research, Mainz, Germany; 7 Preventive Cardiology and Preventive Medicine, Department of Cardiology, University Medical Center of the Johannes Gutenberg-University Mainz, Mainz, Germany; 8 Department of Health Sciences, Applied Health and Social Psychology, Fulda University of Applied Sciences, Fulda, Germany; 9 Department of Ophthalmology, University Medical Center of the Johannes Gutenberg University Mainz, Mainz, Germany; 10 Institute of Medical Biostatistics, Epidemiology and Informatics (IMBEI), University Medical Center of Johannes Gutenberg University, Mainz, Germany; 11 Institute of Clinical Chemistry and Laboratory Medicine, University Medical Center of the Johannes Gutenberg-University Mainz, Mainz, Germany; 12 Metabolic Liver Research Program, University Medical Center of the Johannes Gutenberg University Mainz, Mainz, Germany; 13 Center for Thrombosis and Hemostasis (CTH), University Medical Center of the Johannes Gutenberg University Mainz, Mainz, Germany; 14 Institute of Molecular Biology (IMB), Mainz, Germany; Iranian Institute for Health Sciences Research, ISLAMIC REPUBLIC OF IRAN

## Abstract

**Background:**

Psychological resilience is known as a protective factor against mental health disorders for which valid measures are indispensable. The present work aims to evaluate the Resilience Scale-5 (RS-5) psychometrically, and provide norm values.

**Methods:**

Data from the Gutenberg Health Study (GHS), encompassing 7,496 participants aged 25 to 86, spanning the years 2017 to 2022, was used. Selectivity, item difficulty, internal consistency, construct and factor validity, as well as factorial invariance were tested. Additionally, correlations and associations with depression, anxiety, and sociodemographic factors were determined. Furthermore, norm values were provided.

**Results:**

The RS-5 displayed robust psychometric properties. Participants reported an average resilience score of 28.94 (SD = 5.53, median = 30, *IQR* = 6, range = 5–35), with those aged ≥75 exhibiting the highest resilience levels (M = 30.21, SD = 5.75, median = 32, *IQR* = 7). The RS-5 displayed a very good model fit, affirming measurement invariance across sex and age decades. Construct validity found support through anticipated intercorrelations with related psychological constructs. Significant correlations (p < .001) linked higher resilience with female gender, advanced age, higher education, elevated household income, and diminished psychological distress.

**Conclusion:**

The RS-5 emerged as a reliable and economic instrument for assessing psychological resilience in individuals aged 25 to 86. The study unraveled distinct sociodemographic characteristics significantly tied to resilience levels within this cohort. In contributing recent norm values tailored to the German population, this research enhances the practical applicability of the RS-5 across diverse contexts and enriches our comprehension of the demographic nuances associated with psychological resilience.

## Introduction

The American Psychological Association (APA) defines psychological resilience as “the process and outcome of successfully adapting to difficult or challenging life experiences, especially through mental, emotional, and behavioral flexibility and adjustment to external and internal demands” [1]. Emerging evidence suggests that the resources and skills linked to achieving a more favorable adjustment, such as increased psychological resilience, can be developed and honed through cultivation and practice [1–3]. A meta-analysis reviewing 60 empirical studies to quantitatively determine the association of psychological resilience and mental health [4], showed negative relations with depression, anxiety, or negative affect and positive relations to life satisfaction and positive affect. Psychological resilience was also positively associated with the personality traits extraversion, openness, agreeableness, and conscientiousness, while negative associations with neuroticism were reported [5]. Further, a recent study showed that the self-reported level of psychological resilience was among the most protective factors against mental distress during the COVID-19 pandemic [2]. Evaluating, practicing, and sustaining psychological resilience can be substantial focal points in the treatment and progression of mental health conditions [3], psychological resilience can have substantial consequences for both individuals and public health in several ways. Thus, psychological resilience is acknowledged as a powerful protective health factor [6–8] and has become an important public health issue of recent decades for which valid measures are indispensable [7].

The Resilience scale (RS-25, [9]) and its shorter versions, the Resilience scale- 13 (RS-13,[10]), Resilience scale-11 (RS-11, [11]) and the Resilience scale-5 (RS-5, [12]) are widely used and valid measures to examine psychological resilience. The original 25-item-form (RS-25) with a 2-factor structure was developed by Wagnild and Young [9]. It conceptualized psychological resilience as protective personality trait which facilitates personal adaptation to difficult or challenging life experiences among the two subscales of “personal competence” and “acceptance of self and life”. The subscale “personal competence" scale summarises characteristics such as self-confidence, independence, control, agility and perseverance. The subscale "acceptance of self and life" scale measures characteristics such as adaptability, tolerance and a flexible view of oneself and one’s own path in life. Schumacher et al. [11] evaluated the German version of the RS-25 and confirmed high internal consistency of the RS-25. However, instead of the postulated 2-factor structure of the RS by Wagnild and Young [9], results of the principal component factor analysis with oblique rotation identified only one general factor. Moreover, a 25-item scale were assumed to be of limited used in epidemiological studies which are mostly restricted by time and costs. Thus, the authors developed the unidimensional RS-11 as a valid alternative allowing for economic assessment of resilience in German-speaking general populations. However, Leppert et al. [10] criticized the RS-11 and developed the RS-13 as a revised short version of the RS-25. Further, von Eisenhart Rothe developed the RS-5 as economical alternative to the RS-11, especially for assessment of psychological resilience in vulnerable groups e.g., in older populations. Construct validity for the RS-25 and its shorter versions has been supported by correlations with depression, anxiety, social connectedness, life satisfaction or physical health [9, 11, 12]. Recently, Schmalbach et al. [6] validated and evaluated the psychometric properties of the RS-5 in a representative survey of the German general population. Overall, the authors found a satisfactory model fit of the RS-5 and appropriate item and scale characteristics. However, the results were based on the 11-item version of the resilience scale. Thus, in order to ensure transferability, the authors recommended further validation of the RS-5. Additionally, stratification for several sociodemographic groups showed a distinct pattern of varying psychological resilience levels that, as the authors stated, should be analyzed in future research [6].

Therefore, the present work aimed to validate the RS-5 in a population-based cohort study. Specifically, we aimed (a) to determine psychological resilience measured with the RS-5 in the German general population across age decades, (b) to assess the psychometric properties of the RS-5, including item characteristics, reliability, factorial structure, invariance across sexes and age decades, and construct validity, as well as (c) to provide recent norm values.

## Materials and methods

### Study design and participants

Data were drawn from the ongoing Gutenberg Health Study (GHS). The GHS started in 2007 as a population-based, prospective, observational single-centre cohort study located in the Rhine-Main-Region [13]. The primary endpoints of the study are myocardial infarction and cardiovascular death. Mortality and diseases of the eye, the immune system, cancer, and mental health were defined as additional endpoints. The protocol and documents of the study were approved by the local data safety commissioner and by the ethics committee of the Medical Chamber of Rhineland-Palatinate (reference no. 2018–13720; original vote: December 12, 2017, latest update: September 27, 2021).

The GHS sample was drawn randomly from the local registries of the city of Mainz and the district of Mainz-Bingen, stratified 1:1 for sex and residence and in equal strata across age decades. An age 35 to 74 at baseline was the inclusion criteria. Exclusion criteria were an insufficient knowledge of the German language and physical or mental inability to visit the study centre for investigation. During a 6-hour examination in the study centre, following standard operating procedures and performed by certified medical technical assistants, cardiovascular risk factors and other clinical variables were complemented by a computer-assisted personal interview, laboratory examinations from venous blood samples, blood pressure, and anthropometric measurements. Study investigations were all conducted in line with the Declaration of Helsinki and principles outlined in recommendations for Good Clinical Practice and Good Epidemiological Practice. Written informed consent was obtained from each participant before their inclusion in the study.

The present study used data from the core cohort (participants included since the baseline assessment of 2007 to 2012) and the newly recruited young- and senior cohorts from the GHS ten-year follow-up which started on 21 December 2017 and ended on 25 May 2022. Quality controlled data of *N* = 7,496 participants with 3,853 men (51.40%) and 3,643 women (48.60%) with a mean age of 60,95 years (*SD* = 13.64) were analyzed.

### Measures

#### Resilience

The Resilience Scale-5 (RS-5) [6] was used to measure psychological resilience. The scale consists of five items on one’s psychological resilience (e.g., I am resolved, I retain interest in many things). Response options ranged from 1 (“strongly disagree”) to 7 (“strongly agree”) and were summarized to a sum score (5–35), with higher values indicating a higher degree of psychological resilience. Internal consistency of the RS-5 was previously reported as *α* = .87 [6].

#### Depression symptoms

Symptoms of depression were captured by the nine-item depression module of the Patient Health Questionnaire (PHQ-9) [14, 15] to examine construct validity and replicate previously found negative associations between resilience and depression [e.g.,[12]]. Among nine items, participants were asked about cognitive and somatic symptoms of depression concerning the last two weeks (e.g., loss of interest, loss of/increased appetite, self-perception, ability to concentrate and sleep, energy levels, feeling down or depressed, and suicidal ideation) on a four-point scale ranging from 0 (“not at all”) to 3 (“nearly every day”). Responses were summarized to a sum score (0–27), with higher values indicating higher symptom burden. A cut-off of ≥ 10 was used to indicate clinically relevant symptoms of depression [16]. In the present sample, the PHQ-9 showed good internal consistency (ω = 0.85).

#### Anxiety symptoms

Symptoms of generalized anxiety were assessed by the two-item Generalized Anxiety Disorder Screener (GAD-2) [17]. Like depression symptoms, symptoms of anxiety were used as a measure of construct validity in line with previous research [e.g., [12]]. Participants rated on a four-point scale from 0 (“not at all”) to 3 (“nearly every day”) to what extent they were affected by the feeling of nervousness, anxiety, and the inability to stop or control their worrying over the last two weeks. Answers were added to a sum score (0–6), with higher values indicating higher symptom burden. A cut-off of ≥ 3 was used to indicate clinically relevant symptoms of generalized anxiety [17, 18].

#### Sociodemographic characteristics

Sociodemographic characteristics were assessed via self-report and in the present study coded as sex (male/female), age (in years and decades), education recoded to high school degree (yes/no), occupational status (employed/not employed), retirement (yes/no), partnership (yes/no) and household size. Equivalized income was calculated by dividing a household’s total monthly net income by root of household size and presented in euros. Combining data of education, profession and income, we defined socioeconomic status (SES) according to [19] ranging from 3 (lowest) to 21 (highest) SES.

#### Statistical analysis

Sample characteristics are reported as absolute numbers and percentages for categorical variables and as means with standard deviations for continuous variables.

Means and standard deviations of the RS-5 scale and its items, item difficulty and corrected item-total correlations were determined. Internal consistency of the scale was determined via McDonald’s Omega.

A confirmatory factor analysis (CFA) was used to test the previous proposed one-factor model by von Eisenhart Rothe et al. [12] consisting of the RS-items C, F, G, H, and I. Maximum likelihood estimation were used to account for the significant deviation from normal distribution [20]. To judge model fit, common fit indices were assessed: the Standardized Root Mean Squared Residual (SRMR), the Root Mean Square Error of Approximation (RMSEA), the Comparative Fit Index (CFI), and the Tucker Lewis Index (TLI). A good model fit was indicated by a RMSEA and SRMR of < .050, and a CFI and TLI of >.950. A RMSEA and SRMR of with values between .050 and .080 and a CFI and TLI of at least .900 demonstrate an adequate fit [21, 22].

Factorial invariance across sex and age decades were sequentially tested evaluating the configural (without constraints), the weak (with equal factor loadings across groups), the strong (with equal factor loadings and item intercepts across groups), as well as the strict invariance (with equal factor loadings, item intercepts and residual variances across groups) of the model as proposed by Meredith and Teresi’s [23]. Each model was compared against the more stringent model. The commonly used method for assessing overall model fit is the chi-square test. As its accuracy is influenced by sample size, potentially leasing to the rejection of reasonable models in cases of large sample sizes, the abovementioned four fit indices were used to compare model fits instead of the chi-square test. Measurement invariance is given if the difference between the models does not surpass the .01 cut-off [24–26].

To test construct validity, group differences for the RS-5 average score with respect to sociodemographic characteristics were tested using t-tests and Tukey’s HSD. Further, intercorrelations of the RS-5 with the related psychological constructs of depression (PHQ-9) and generalized anxiety (GAD-2) were investigated. Intercorrelations were analysed as Spearman correlations. P-values were assumed to be significant with *p*< .001. Additionally, associations with sociodemographic characteristics and related psychological constructs were determined by multiple regression analysis.

Normative values for the RS-5 were calculated using percentiles for the full sample and for subsamples based on age and sex.

Statistical analyses were performed using the statistical program R (version 4.2.1, packages: dplyr [27], psych [28], lavaan [29]).

## Results

### Participants characteristics

Data from 7,496 participants at ten-year follow-up with a mean age of 60.65 (*SD* = 13.64; range = 25–86 years) were analysed. Of those, 3,643 (48.60%) were female. The average psychological resilience score of the total sample was, with participants aged ≥ 75 years reporting the highest level of psychological resilience (*M* = 30.21, *SD* = 5.75, median = 32, *IQR* = 7). For detailed information on participants characteristics, see Table 1.

**Table 1:** Characteristics of the participants (N = 7,496).

Sociodemographic factors			
Sex (*N*, %)	Total (*N* = 7,496)	Men (*N* = 7,496)	Women (*N* = 7,496)
*Men*	3,853 (51.40%)		
*Women*	3,643 (48.60%)		
Age in years (*M*, *SD*)	60.65 (13.64)	61.45 (13.71)	59.81 (13.51)
Age decades (*N*, %)			
*25–34 years*	364 (4.86%)	176 (4.57%)	188 (5.16%)
*35–44 years*	583 (7.78%)	271 (7.03%)	312 (8.56%)
*45–54 years*	1,457 (19.44%)	702 (18.22%)	755 (20.72%)
*55–64 years*	1,912 25.51%)	960 (24.92%)	952 (26.13%)
*65–74 years*	1,739 (23.20%)	924 (23.98%)	815 (22.37%)
*≥ 75 years*	1,441 (19.22%)	820 (21.28%)	621 (17.05%)
High school degree (*N*, %)			
*Yes*	2,413 (37.74%	1,266 (38.94%)	1,147 (36.51%)
*No*	3,980 (62.26%)	1,985 (61.06%)	1,995 (63.50%)
Occupational status (*N*, %)			
*Employed (yes)*	3,920 (56.73%)	2,043 (56.08%)	1,877 (57.45%)
*Retired (yes)*	2,990 (43.27%)	1,600 (43.92%)	1,390 (42.55%)
Partnership, (*N*, %)			
*Yes*	5,225 (71.38%)	2,843 (75.31%)	2,382 (67.19%)
*No*	2,095 (28.62%)	932 (24.69%)	1,163 (32.81%)
Household size (*N*, *%*)			
*1*	1,240 (17.15%)	521 (13.99%)	719 (20.52%9
*2*	4,105 (56.79%)	2,185 (58.66%)	1,920 (54.79%)
*3*	920 (12.73%)	507 (13.61%)	413 (11.79%)
*≥ 4*	964 (13.34%)	512 (13.74%)	452 (12.91%)
Equivalized income in euros, (*M*, *SD;* median, *IQR*)	2,889.58 (1,934.37)	3,063.17 (2,101.34)	2,697.22 (1,710.48)
	2,453 (1,495)	2,625 (1,475)	2,375 (1,438)
SES (*M*, *SD*)	14.10 (4.20)	14.76 (4.15)	13.40 (4.14)
**Mental health**			
Symptoms of depression (*M*, *SD; median*, *IQR*)	3.65 (3.43)	3.25 (3.24)	4.09 (3.58)
	3 (4)	2 (4)	3 (4)
Clinical relevant symptoms of depression (*N*, %)			
*Yes*	456 (6.27%)	191 (5.07%)	265 (7.57%)
*No*	6,811 (93.73%)	3,575 (94.93%)	3,236 (92.43%)
Symptoms of generalized anxiety (*M*, *SD; median*, *IQR*)	0.95 (1.17)	0.75 (1.05)	1.16 (1.26)
	1 (2)	0 (1)	1 (2)
Clinical relevant symptoms of anxiety (*N*, %)			
*Yes*	616 (8.38%)	219 (5.76%)	397 (11.18%)
*No*	6,733 (91.62%)	3,580 (94.24%)	3,153 (88.81%)
Psychological resilience (*M*, *SD; median*, *IQR*)	28.94 (5.53)	28.71 (5.48)	29.18 (5.57)
	30 (6)	30 (5)	30 (6)

*Note*. Occupational status “employed” summarizes full-time, part-time, and irregular employment; *M* = Mean; *SD* = Standard deviation; *IQR* = Interquartile range.

### Reliability and item characteristics

Internal consistency for the RS-5 was *ω* = .90. Means (M) and standard deviations (SD) for the items as well as item difficulties (P_i_) and corrected item-total correlation (r_it_) were shown in Table 2. Participants scored highest on the item C lowest on the item F. The item difficulties varied between .78 and .87. The corrected item-total correlations achieved very satisfactory values of r_it_ = .64 to r_it_ = .80. The corrected item-total correlations of all items satisfied the common cut-off point of >.50 [30].

**Table 2:** Means (M), standard deviations (SD), median and interquartile range (IQE), item difficulties (P_i_) and corrected item-total correlations (r_it_) for the RS-5.

Item								
	English	German	*M*	*SD*	*median*	*IQR*	*P*_*i*_	*r*_*it*_
C	Keeping interested in things is important to me.	Es ist mir wichtig, an vielen Dingen interessiert zu bleiben.	6.09	1.36	7	1	0.87	0.71
F	I am determined.	Ich bin entschlossen.	5.47	1.41	6	2	0.78	0.69
G	I keep interested in things.	Ich behalte an vielen Dingen Interesse.	5.86	1.30	6	2	0.83	0.80
H	I can usually find something to laugh about.	Ich finde öfter etwas, worüber ich lachen kann.	5.75	1.40	6	2	0.82	0.64
I	I can usually look at a situation in a number of ways.	Normalerweise kann ich eine Situation aus mehreren Perspektiven betrachten.	5.78	1.31	6	2	0.83	0.68

### Factor validity and factorial invariance

Overall, results of the CFA confirmed a good model fit. All assessed fit indices indicated an adequate to very good model fit except for the RMSEA which was greater than .08 (CFI = .958; TLI = .917; SRMR = .035; RMSEA = .146, 90% confidence interval [.134 –.158]). Although the RMSEA surpasses the proposed cut-off of .08, the CFI is more robust and less susceptible to larger samples. Thus, Byrne [31] and Byrne and Stewart [32] have suggested to mostly use the CFI as a fit indicator. Since the CFI yields a good result, we assume our model to have a good fit. Factor loadings were between .67 and .90.

The CFI differences were well below the cut-off value of .01. Therefore, strict factorial invariance across sex and age decades could be shown. Detailed results of the analysis of factorial invariance across sex and age decades are depicted in S1 Table.

### Construct validity

The RS-5 score differed statistically significantly with *p* < .001 between age decades. Participants aged ≥ 75 years reported the highest degree of psychological resilience compared to participants of the remaining five younger age decades. Further, statistically different RS-5 average scores were found between men and women, *t*(7455.2) = -3.65, *p* < .001. Higher psychological resilience was found in women compared to men. No differences were obtained in participants with and without a partner. Groups of education levels differed statistically significantly, *t*(5586.9) = -4.73, *p* < .001. Participants having a high school degree reported higher psychological resilience compared to participants without a high school degree. So did employed and retired participants, *t*(6222.8) = -8.88, *p* < .001. Employed participants mentioned significant lower psychological resilience than retired ones. Complete group comparison results, including means and standard deviations for all groups, can be found in Table 3.

**Table 3:** Group-differences in psychological resilience (RS-5).

	*N*	*M*	*SD*	*median*	*IQR*	*t*	*95%-Confidence intervall*	*p-value*
Age decades (Ref. ≥ 75 years)								
*25–34 years*	364	27.52	4.72	28	6	2.68	1.77–3.60	< .001
*35–44 years*	583	27.94	4.72	29	5	2.27	1.51–3.04	< .001
*45–54 years*	1,457	28.25	5.3	29	5	1.95	1.37–2.53	< .001
*55–64 years*	1,912	28.77	5.57	30	6	1.44	0.89–1.98	< .001
*65–74 years*	1,739	29.28	5.66	30	6	0.93	0.37–1.48	< .001
Sex						-3.65		< .001
*Men*	3,853	28.71	5.48	30	5			
*Women*	3,643	29.18	5.57	30	6			
Partner						-2.93		= .003
*Yes*	5,225	29.06	5.41	30	6			
*No*	2,095	28.63	5.68	30	7			
Education						-4.73		< .001
*High school degree*	2,413	29.13	5.02	30	5			
*No high school degree*	3,980	28.49	5.70	30	6			
Occupational status						-8.88		< .001
*Employed*	3,920	28.45	5.32	30	6			
*Retired*	2,990	29.63	5.65	31	7			

*Note*. Employed summarizes full-time, part-time and irregular employment; *M* = Mean; *SD* = Standard deviation; *IQR* = Interquartile range; *t* = size of the difference relative to the variation in sample.

Low but substantial negative intercorrelations of the RS-5 with the continuous and binary variable of PHQ-9, indicative of depressive symptom burden, as well as with the continuous and binary variable of GAD-2, indicative of generalized anxiety symptom burden, were found, suggesting construct validity. As shown in Table 4, a higher RS-5 score was associated with a lower PHQ-9 score and clinically relevant symptoms of depression, as well as with a lower score on the GAD-2 and clinically relevant symptoms of generalized anxiety.

**Table 4:** Correlation coefficients between the RS-5 and related psychological constructs: Depression (PHQ-9) and anxiety (GAD-2).

Psychological scales	RS-5	PHQ-9	GAD-2
RS-5	1.00		
PHQ-9	-.33*** (-.20***)	1.00	
GAD-2	-.25*** (-.17***)	.60*** (.46***)	1.00

*Note*. Correlation coefficients between the binary variables of PHQ-9 and GAD-2 and the RS-5 are shown in brackets, ***p < .001

Additionally, a multiple regression analysis including sex, age, high school degree, occupational status partnership, household size, equivalized household income, and symptoms of depression, as well as generalized anxiety as explanatory variables was performed. Female sex, increasing age, having a high school degree, a higher equivalized household income, and less psychological distress were found to be associated with higher levels of psychological resilience. For detailed results, see Table 5.

**Table 5:** Multiple regression analysis with RS-5 as outcome (N = 5,354).

Model fit	F (9,5344) = 72.87, p < .001; R^2^ = .109; R^2^_adj._ = .108			
	*B*	*SE*	*95%- Confidence intervall*	*p-value*
Sex (female)	.96	.14	.68–1.24	< .001
Age	.04	.01	.02-.06	< .001
High school degree (yes)	.79	.15	.50–1.09	< .001
Occupational status (retired)	.39	.21	-.04-.81	.08
Partner (yes)	-.25	.18	-.62-.12	.18
Household size	.09	.01	-.06-.25	.23
Equivalized household income	.00	.00	.00-.00	< .001
Symptoms of depression	-.36	.03	-.42—.30	< .001
Symptoms of anxiety	-.34	.01	-.51–0.16	< .001

*Note*. Significant results marked in bold. Varying *N* compared to full sample due to missingness in explanatory variables; *B =* unstandardized coefficient; *SE =* standard error.

### Norm values

Table 6 presents the norm values stratified by sex and age group (<60/ ≥60 years) for the RS-5. Because the RS-5 sum score deviated from a normal distribution, score distribution was reported using percentiles.

**Table 6:** Percentiles for the RS-5 according to sexes and age decades.

	Total (*N* = 7,496)		Men (N = 3,853)		Women (N = 3,643)	
Age group	<60 years	≥60 years	<60 years	≥60 years	<60 years	≥60 years
RS-5 sum score						
1	0	0	0	0	0	0
2	0	0	0	0	0	0
3	0	0	0	0	0	0
4	0	0	0	0	0	0
5	.4	.8	.4	.7	.4	.8
6	.6	1.0	.6	1.0	.7	1.0
7	.7	1.3	.6	1.4	.8	1.3
8	1.0	1.5	.9	1.7	1.0	1.4
9	1.3	1.7	1.4	1.9	1.3	1.6
10	1.6	2.1	1.5	2.3	1.6	1.8
11	1.9	2.5	1.9	2.9	1.9	2.0
12	2.2	2.7	2.2	3.1	2.3	2.2
13	2.5	2.9	2.6	3.4	2.5	2.3
14	2.7	3.1	2.7	3.5	2.6	2.6
15	3.1	3.5	3.2	3.9	3.0	3.1
16	3.6	3.9	3.6	4.2	3.6	3.5
17	4.2	4.3	4.3	4.6	4.1	3.9
18	5.1	4.7	5.1	5.1	5.1	4.3
19	5.9	5.4	5.8	5.6	6.0	5.3
20	7.3	7.2	6.9	7.1	7.8	7.2
21	9.0	8.1	8.6	8.0	9.4	8.3
22	11.2	9.5	11.0	9.6	11.4	9.5
23	14.2	11.7	13.9	11.5	14.5	11.8
24	17.6	13.8	17.0	13.8	18.1	13.7
25	22.5	17.0	22.4	17.8	22.7	16.0
26	28.2	21.0	28.2	22.2	28.1	19.7
27	35.6	25.7	36.4	27.9	34.8	23.2
28	43.5	30.5	44.9	33.0	42.2	27.6
29	52.3	37.8	54.1	40.7	50.6	34.5
30	63.6	47.6	66.3	51.7	61.0	42.9
31	73.6	56.7	76	61.1	71.2	51.8
32	82.0	64.7	84.9	69.5	79.2	59.2
33	88.3	73.2	90.1	77.4	86.6	68.4
34	92.9	79.6	93.9	83.7	91.8	75.1
35	100	100	100	100	100	100
n	3,404	4,092	1,678	2,175	1,726	1,917

## Discussion

Psychological resilience, as a protective factor against mental health disorders, has become an important public health issue of recent decades for which valid measure are indispensable. The RS-5 is a widely used and valid measure to assess psychological resilience. However, the latest available psychometric properties of the RS-5 were based on the RS-11. Thus, to rule out any external influences on the measurement, further validation of the RS-5 has been recommended. The present work aimed to validate and standardize the RS-5 and to report recent norm values for the German general population.

Highest level of psychological resilience was reported by participants aged 75 years and older. The RS-5 item characteristics and internal consistency were found to be satisfactory. All factor loadings were above .50, attesting to the previously proposed one-factor model [6, 12]. Further, fit indices of the CFA indicated an adequate to very good model fit except the RMSEA. However, the CFI has been suggested to mostly be used as fit indicator since it is more robust and less susceptible to larger samples [31, 32]. Thus, the RS-5 appears to be a valid measure to assess psychological resilience in German-speaking populations. Additionally, strict measurement invariance for sex and age decades was given, indicating the ability to statistically compare men and women and individuals of different age decades. Group comparisons regarding sociodemographic characteristics showed statistically significant differences in psychological resilience (*p*< .001). These results were partly comparable to previously reported sociodemographic characteristics and RS-5 differences. Higher psychological resilience was found among participants having a high school degree, replicating previous findings [6]. In contrast to Schmalbach et al. [6], in the present sample, higher psychological resilience was reported by women compared to men and by participants who were retired. No statistically significant differences between participants with and without partners were found. Study design, sample, or region differences may account for the contrary findings. Lampert and Koch-Gromus [33] pointed to significant regional differences regarding social parameters (e.g., occupational status) that exist within Germany, which contribute to health and life expectancy disparities. While Schmalbach et al. [6] used data from a representative nationwide survey sample. Data for the present work was drawn from a population-based cohort study of the metropolitan Rhine-Mine region in western Mid-Germany. Therefore, the GHS may constitute a wealthier and more homogenous sample with a higher education level than the sample of Schmalbach et al. [6]. Negative intercorrelations with mental distress supported construct validity. In line with previous research (e.g., [4, 6]) negative correlations with symptoms of depression (PHQ-9) and general anxiety (GAD-2) were found. Moreover, a multiple regression analysis showed positive associations between female sex, increasing age, high school degree, and a higher equivalized household income with psychological resilience. Longitudinal studies on predictors of psychological resilience were recommended to identify important influencing factors and vulnerable subgroups. Extensive epidemiological research consistently underscores the inverse relationship between psychological resilience and the incidence of mental health outcomes. Individuals with higher levels of psychological resilience exhibit a lower susceptibility to psychopathologies, suggesting a protective role in maintaining mental health [34, 35]. Longitudinal studies examining individuals over time reinforce this association, revealing that heightened psychological resilience is linked to lower rates of chronic stress-related illnesses. This extends beyond mental health, encompassing a spectrum of conditions, including cardiovascular diseases, autoimmune disorders, and psychiatric ailments [36, 37]. Recognizing psychological resilience as a multidimensional protective factor emphasizes its significance in promoting mental health and well-being in facing life’s adversities.

Finally, recent norm values across sex and age decades for the German population were provided.

Despite the great strength of the present work using data from a large population-based cohort study covering a wide age range, results should be interpreted concerning study’s limitations. Selective participation might limit the generalizability of the results using data from the GHS ten-year follow-up. Hence, the results based on a non-representative sample of the German population, the provided norms may have limited utility. However, the results will serve as a useful comparison for other studies using RS-5 in German, Further, the present findings represent individuals of the German population; comparisons can only be made for Western demographics. Thus, cross-culturally, psychometric evaluation of the RS-5 is warranted. We recommended to expand the present results by further determining validity and reliability of the RS-5, in particular regarding domains we were not able to address with the present study design. For instance, due to the cross-sectional study design, test-retest reliability or predictive validity could not be determined. Therefore, future research assessing test-retest reliability is desirable. Further, as psychological resilience is defined as a trait by the RS-5, it is supposed to show higher test-retest correlation that of state measures (e.g., depression) and comparable to other trait measures (e.g., Big Five). Construct validity was determined by group differences for the RS-5 average score with respect to sociodemographic characteristics and intercorrelations of the RS-5 with two screeners of related psychological constructs (PHQ-9 and GAD-2). Analysing the associations with other constructs, e.g., in a nomological net using a wider variety of measures, especially multi-item measures, would be also add important information about its construct validity. Psychometric properties may also change when the scale is applied to different samples (e.g., clinical populations).

Summed up, item and construct properties and the reliability of the RS-5 were found to be satisfactory. A CFA supported the unidimensional factorial structure, while measurement invariance could be confirmed across sex and age decades. Thus, the RS-5 offers a valid and economical screening measure, especially in large-scale population studies, which are mostly restricted in time and cost. Future research efforts should prioritize the identification of specific neurobiological, psychosocial, and genetic correlates of psychological resilience, as a deeper understanding of these factors is pivotal in informing targeted preventive approaches. Such insights hold relevance in mental health and well-being, where psychological resilience emerges as a key factor in mitigating the impact of stressors and adversity.

## Supporting information

S1 Table Results of the analysis of factorial invariance across sexes and age decades. (DOCX)

## References

[1] Resilience: American Psychological Association; `Available from: https://www.apa.org/topics/resilience.

[2] Gilan D, Mussig M, Hahad O, Kunzler AM, Samstag S, Rothke N, et al. Protective and Risk Factors for Mental Distress and Its Impact on Health-Protective Behaviors during the SARS-CoV-2 Pandemic between March 2020 and March 2021 in Germany. Int J Environ Res Public Health. 2021;18(17). doi: 10.3390/ijerph18179167 34501756 PMC8431087

[3] Chmitorz A, Kunzler A, Helmreich I, Tuscher O, Kalisch R, Kubiak T, et al. Intervention studies to foster resilience—A systematic review and proposal for a resilience framework in future intervention studies. Clin Psychol Rev. 2018;59:78–100. doi: 10.1016/j.cpr.2017.11.002 29167029

[4] Hu T, Zhang D, Wang J. A meta-analysis of the trait resilience and mental health. Personality and Individual Differences. 2015;76:18–27.

[5] Oshio A, Taku K, Hirano M, Saeed G. Resilience and Big Five personality traits: A meta-analysis. Personality and Individual Differences. 2018;127:54–60.

[6] Schmalbach B, Zenger M, Strauß B, Hinz A, Steffens-Guerra I, Decker O, et al. Validation and psychometric properties of the resilience scale-5 (RS-5) results of a representative survey of the German general population. Health Science Journal,. 2016;10(5):1.

[7] Leppert K, Gunzelmann T, Schumacher J, Strauß B, Brähler E. Resilienz als protektives Persönlichkeitsmerkmal im Alter. Psychother Psychosom Med Psychol. 2005;55(08):365–9. 16049872 10.1055/s-2005-866873

[8] Farber F, Rosendahl J. Trait resilience and mental health in older adults: A meta-analytic review. Personal Ment Health. 2020;14(4):361–75. doi: 10.1002/pmh.1490 32573068

[9] Wagnild GM, Young HM. Development and psychometric evaluation of the Resilience Scale. J Nurs Meas. 1993;1(2):165–78. 7850498

[10] Leppert K, Koch B, Brähler E, Strauß B. Die resilienzskala (RS)–Überprüfung der langform RS-25 und einer kurzform RS-13. Klinische Diagnostik und Evaluation. 2008;1(2):226–243.

[11] Schumacher J, Leppert K, Gunzelmann T, Strauß B, Brähler E. Die Resilienzskala—Ein Fragebogen zur Erfassung der psychischen Widerstandsfähigkeit als Persönlichkeitsmerkmal. Z Klin Psychol, Psychiatrie, Psychotherapie. 2005;53:16–39.

[12] von Eisenhart Rothe A, Zenger M, Lacruz ME, Emeny R, Baumert J, Haefner S, et al. Validation and development of a shorter version of the resilience scale RS-11: results from the population-based KORA-age study. BMC Psychol. 2013;1(1):25. doi: 10.1186/2050-7283-1-25 25566373 PMC4270035

[13] Wild PS, Zeller T, Beutel M, Blettner M, Dugi KA, Lackner KJ, et al. Die Gutenberg Gesundheitsstudie. Bundesgesundheitsblatt—Gesundheitsforschung—Gesundheitsschutz. 2012;55(6–7):824–30. doi: 10.1007/s00103-012-1502-7 22736163

[14] Kroenke K, Spitzer RL, Williams JB. The PHQ‐9: validity of a brief depression severity measure. Journal of general internal medicine. 2001;16(9):606–13. doi: 10.1046/j.1525-1497.2001.016009606.x 11556941 PMC1495268

[15] Martin A, Rief W, Klaiberg A, Braehler E. Validity of the Brief Patient Health Questionnaire Mood Scale (PHQ-9) in the general population. Gen Hosp Psychiatry. 2006;28(1):71–7. doi: 10.1016/j.genhosppsych.2005.07.003 16377369

[16] Kocalevent RD, Hinz A, Brahler E. Standardization of the depression screener patient health questionnaire (PHQ-9) in the general population. Gen Hosp Psychiatry. 2013;35(5):551–5. doi: 10.1016/j.genhosppsych.2013.04.006 23664569

[17] Löwe B, Wahl I, Rose M, Spitzer C, Glaesmer H, Wingenfeld K, et al. A 4-item measure of depression and anxiety: validation and standardization of the Patient Health Questionnaire-4 (PHQ-4) in the general population. J Affect Disord. 2010;122(1–2):86–95. doi: 10.1016/j.jad.2009.06.019 19616305

[18] Wicke FS, Krakau L, Lowe B, Beutel ME, Brahler E. Update of the standardization of the Patient Health Questionnaire-4 (PHQ-4) in the general population. J Affect Disord. 2022;312:310–4. doi: 10.1016/j.jad.2022.06.054 35760191

[19] Lampert T, Kroll LE. Die Messung des sozioökonomischen Status in sozialepidemiologischen Studien. In: Richter M, Hurrelmann K, editors. Gesundheitliche Ungleichheit: VS Verlag für Sozialwissenschaften; 2009.

[20] Chou CP, Bentler PM, Satorra A. Scaled test statistics and robust standard errors for non-normal data in covariance structure analysis: a Monte Carlo study. Br J Math Stat Psychol. 1991;44 (Pt 2):347–57. doi: 10.1111/j.2044-8317.1991.tb00966.x 1772802

[21] Lt Hu, Bentler PM. Cutoff criteria for fit indexes in covariance structure analysis: Conventional criteria versus new alternatives. Structural Equation Modeling: A Multidisciplinary Journal. 1999;6(1):1–55.

[22] van de Schoot R, Lugtig P, Hox J. A checklist for testing measurement invariance. European Journal of Developmental Psychology. 2012;9(4):486–92.

[23] Meredith W, Teresi JA. An essay on measurement and factorial invariance. Med Care. 2006;44(11):69–77. doi: 10.1097/01.mlr.0000245438.73837.89 17060838

[24] Chen FF. Sensitivity of Goodness of Fit Indexes to Lack of Measurement Invariance. Structural Equation Modeling: A Multidisciplinary Journal. 2007;14(3):464–504.

[25] Cheung GW, Rensvold RB. Evaluating Goodness-of-Fit Indexes for Testing Measurement Invariance. Structural Equation Modeling: A Multidisciplinary Journal. 2002;9(2):233–55.

[26] Hirschfeld G, von Brachel R. Improving Multiple-Group confirmatory factor analysis in R–A tutorial in measurement invariance with continuous and ordinal indicators. Practical Assessment, Research, and Evaluation. 2014;19(1):7.

[27] Wickham H, François R, Henry L, Müller K, Vaughan D. dplyr: A Grammar of Data Manipulation. 2023.

[28] Revelle W. psych: Procedures for Psychological, Psychometric, and Personality Research. 2021.

[29] Rosseel Y. “lavaan: An R Package for Structural Equation Modeling.”. Journal of Statistical Software. 2012;48(2):1–36.

[30] Hair J, Black W, Babin B, Anderson R. Multivariate Data Analysis. 2010; Prentice Hall, Upper Saddle River, NJ.

[31] Byrne BM. Testing for Multigroup Invariance Using AMOS Graphics: A Road Less Traveled. Structural Equation Modeling: A Multidisciplinary Journal. 2004;11(2):272–300.

[32] Byrne BM, Stewart SM. TEACHER’S CORNER: The MACS Approach to Testing for Multigroup Invariance of a Second-Order Structure: A Walk Through the Process. Structural Equation Modeling: A Multidisciplinary Journal. 2006;13(2):287–321.

[33] Lampert T, Koch-Gromus U. Soziale Ungleichheit und Gesundheit. Bundesgesundheitsblatt Gesundheitsforschung Gesundheitsschutz. 2016;59(2):151–2. doi: 10.1007/s00103-015-2306-3 26781779

[34] Masten AS. Global perspectives on resilience in children and youth. Child Dev. 2014;85(1):6–20. doi: 10.1111/cdev.12205 24341286

[35] Bonanno GA. Loss, trauma, and human resilience: have we underestimated the human capacity to thrive after extremely aversive events? Am Psychol. 2004;59(1):20–8. doi: 10.1037/0003-066X.59.1.20 14736317

[36] Connor KM, Davidson JR. Development of a new resilience scale: the Connor-Davidson Resilience Scale (CD-RISC). Depress Anxiety. 2003;18(2):76–82. doi: 10.1002/da.10113 12964174

[37] Seery MD, Holman EA, Silver RC. Whatever does not kill us: cumulative lifetime adversity, vulnerability, and resilience. J Pers Soc Psychol. 2010;99(6):1025–41. doi: 10.1037/a0021344 20939649

