# Supplementary material for: Assessment of psychological resilience in a large cohort of the general population: Validation and norm values of the RS-5
Source: PLoS One. 2024 Oct 11;19(10):e0309197. doi: 10.1371/journal.pone.0309197 (PMC11469496; doi:10.1371/journal.pone.0309197)
Supplement: S1 Table — (DOCX) [file pone.0309197.s001.docx]

**S1 Table. Results of the analysis of factorial invariance across sexes and age decades.**

|  | △ CFI | △ TLI | △ RMSEA | △ SRMR |
| --- | --- | --- | --- | --- |
| Model -sex |  |  |  |  |
| Configural |  |  |  |  |
| Weak | -0.001 | 0.022 | -0.021 | 0.003 |
| Strong | -0.003 | 0.01 | -0.011 | 0.003 |
| Strict | -0.003 | 0.008 | -0.01 | 0.005 |
| Model -age decades |  |  |  |  |
| Configural |  |  |  |  |
| Weak | -0.003 | 0.027 | -0.028 | 0.009 |
| Strong | -0.014 | 0.002 | -0.002 | 0.011 |
| Strict | -0.021 | -0.001 | 0.002 | 0.009 |
